# From clinical phenotypes to cellular mechanisms: a precision medicine framework for COPD

**DOI:** 10.3389/fimmu.2026.1846920

**Published:** 2026-05-21

**Authors:** Jianjun Wu, Xiaomeng Cheng, Fangyuan Yu, Ruitao Wang, Benzhang Zhao, Hantao Zhao, Wenjuan Zhang, Yufei Chen

**Affiliations:** Respiratory Department, The Third Affiliated Hospital, Beijing University of Chinese Medicine, Beijing, China

**Keywords:** cellular phenotypes, chronic obstructive pulmonary disease, clinical phenotypes, management strategy, precision medicine

## Abstract

Chronic obstructive pulmonary disease (COPD) is a persistent, often progressive lung disease with a highly heterogeneous patient population. Its management has traditionally relied on clinical symptoms and pulmonary function tests. However, this approach has limitations due to a poor alignment between the clinical symptoms and underlying individual pathological mechanisms, as well as a heavy reliance on chest imaging, restricting its wide application in resource-limited settings. This review aimed to bridge the gap between clinical presentation and underlying mechanisms by systematically mapping cellular inflammatory phenotypes to targeted therapies. We critically examined the evolution from clinical to cellular phenotyping, highlighting the potential of mechanistic, cell-based classifications to improve COPD management. The proposed cellular phenotype classification—neutrophilic (>60% sputum neutrophils), eosinophilic (≥3% eosinophils), lymphocytic, macrophage, and mixed granulocytic—has been validated in multinational cohorts and provides a framework for precision interventions: inhibiting the CXCR1/2 pathway or neutrophil elastase for the neutrophilic phenotype, and anti-interleukin-5/interleukin-13 biologics or Th2 blockade for the eosinophilic phenotype. We propose a four-tiered diagnostic pathway comprising biomarker screening, multi-omics validation, consensus assignment, and dynamic therapy escalation. This approach shifts COPD management from symptom control to pathogenesis modification. However, substantial challenges remain in translating cellular phenotypes into routine practice, warranting further research.

## 1 Introduction

Chronic obstructive pulmonary disease (COPD) is a heterogeneous illness (1, 2). Affected patients can present with various clinical symptoms (3), physical complaints, comorbidities (4), laboratory findings (5), and treatment outcomes (6). Therefore, the appropriate classification of COPD patients is essential for guiding treatment decisions, stratifying the exacerbation risk, and predicting patient prognosis. This has led to the concept that phenotyping should precede treatment initiation (7–9).

The causes of COPD are similarly diverse. Beyond tobacco, factors like biomass smoke, air pollution, early-life events, and genetic susceptibility such as alpha-1 antitrypsin deficiency can all trigger distinct inflammatory pathways, shaping very different disease trajectories.

There exist several different COPD classification systems, based on patient clinical, imaging, physiological, and molecular phenotypes (2). In particular, the phenotype-centered approach is often used in clinical practice. In 2010, Han et al. proposed an initial concept focused on COPD phenotypes and the classification of COPD patients into different subgroups based on their clinical presentations, symptom patterns, exacerbation risks, treatment responses, disease progression, and mortality (8). In 2012, the first personalized COPD treatment protocol was pioneered in Spain, based on patient clinical phenotypes and comprising: 1) non-frequent exacerbations with chronic bronchitis or emphysema, 2) COPD–asthma overlap, 3) frequent exacerbations with emphysema predominance, and 4) frequent exacerbations with chronic bronchitis predominant phenotypes (10). In the same year, Czechoslovakian COPD guidelines introduced six primary clinical phenotypes, comprising: 1) frequent exacerbations, 2) COPD–asthma overlap, 3) COPD-bronchiectasis overlap, 4) emphysema, 5) bronchitis, and 6) pulmonary cachexia phenotypes (11). The Global Initiative for COPD (GOLD) is another classification framework that categorizes COPD patients according to their pulmonary function test results and clinical symptom burden evaluations. GOLD offers greater practicality than imaging-dependent guidelines in resource-limited settings (11, 12). All these classification systems have been used as a basis for developing the phenotype-specific management approach (10, 11), which has gained increasing popularity in clinical practice (13). However, this phenotype-based COPD management approach has limitations, including a lack of standardization in phenotype definitions, phenotypic instability over time, and inability of phenotypes to fully capture underlying biological mechanisms (14).

This review aims to critically examine how cellular phenotyping can build on clinical classification to link COPD heterogeneity more directly to targeted therapies, thereby advancing precision medicine in COPD.

## 2 Search strategy

A targeted literature search of PubMed and Embase was conducted to identify studies (up to December 2025) on COPD phenotypes, their underlying inflammatory mechanisms, and related targeted therapies. As this is a narrative review rather than a systematic review, a formal PRISMA-based article count was not performed. Search terms combined key concepts for clinical phenotypes, cellular endotypes, and specific pathways or treatments. Priority was given to phase III clinical trials and multinational cohort validation studies. References of retrieved articles and major guidelines were also screened.

## 3 Overview of clinical phenotypes

### 3.1 Chronic bronchitis phenotype

Compared to patients with non-chronic bronchitis phenotypes, those with the chronic bronchitis phenotype commonly had more severe dyspnea, poorer lung function, greater airway wall thickening, and a higher acute exacerbation frequency (12, 15). Meanwhile, compared to patients with the emphysema phenotype, patients with the chronic bronchitis phenotype demonstrated worse nocturnal symptoms and sleep apnea, as well as a higher risk of an overweight status and cardiovascular comorbidities (16, 17), poorer quality of life, and elevated anxiety/depression levels (18).

These patients can benefit from phosphodiesterase-4 inhibitors such as roflumilast (19, 20), Myrtol (ELOM-080) (11), and mesenchymal stem cell therapy. These interventions reduce exacerbation frequency (11, 19, 20), alleviate cough and sputum production (11), and delay lung function deterioration (20).

### 3.2 Emphysema phenotype

Compared to patients with CT-defined airway-dominant phenotypes, those with the emphysema-predominant phenotype were reported to have a lower body mass index (BMI), more severe airflow obstruction, and shorter 6-minute walk distance (21). Relative to patients with chronic bronchitis, those patients exhibited higher autoantibody titers (22) and an increased prevalence of cardiovascular disease (23). Progressive emphysema correlated with accelerated forced expiratory volume in 1 second (FEV_1_) and BMI decline, higher exacerbation rates, and increased mortality (24).

Selected patients benefit from bronchoscopic lung volume reduction, particularly endobronchial valve placement, and surgical lung volume reduction, both improving lung function, exercise tolerance, and quality of life (25, 26).

### 3.3 Frequent exacerbation phenotype

Compared to patients with non-frequent exacerbations, COPD patients with frequent exacerbation phenotypes experienced a faster decline in outdoor activity time (27), increased fatigue, poorer quality of life, worse lung function, accelerated FEV_1_ reduction (1, 28), higher mortality, elevated future exacerbation risks, decreased sputum microbiome α-diversity and increased β-diversity (29), and more extrapulmonary comorbidities (e.g., cardiovascular disease and osteoporosis) (30).

Roflumilast reduced exacerbation frequency in this subgroup, transitioning patients from frequent to stable non-frequent exacerbation states (31). Also, inhaled corticosteroid (ICS) combined with bronchodilators further decreased exacerbation rates and improved the quality of life of patients (32). The role of azithromycin remains unclear (33, 34), and ongoing RELIANCE trial may further clarify its efficacy in preventing disease progression.

### 3.4 COPD–asthma overlap phenotype

This phenotype features incompletely reversible obstruction, a marked bronchodilator response, elevated blood eosinophils and FeNO, and often an atopic background that sustains type 2 inflammation. Compared to patients with asthma or COPD alone, these patients suffered more frequent acute exacerbations, higher hospitalization incidence, poorer quality of life, and faster lung disease progression (35).

Potential therapies include TSLP inhibitors, interleukin-33 blockade (33), interleukin-17 inhibition (34), triple therapy (36), and omalizumab, with benefits primarily observed in lung function improvement.

## 4 COPD as a cellular inflammation-driven disorder: benefits of identifying and targeting cellular phenotypes

COPD is characterized by chronic airway inflammation and predominantly involves different cell types, such as macrophages, neutrophils, Tc1, Th1, Th17, and ILC3 lymphocytes (37, 38). These cells and their relevant inflammatory mediators can drive COPD pathogenesis, directly determining the patient’s symptoms, clinical signs, and pathophysiology. Classifying COPD patients into distinct cellular phenotypes and tailoring therapies to cell-specific inflammatory pathways may thus offer clinical benefits.

In several clinical studies, induced sputum and blood examination, as a non-invasiveness and easily accessible test, was selected as the primary method to directly reflect airway inflammation for cellular phenotype classification (39, 40). The cutoffs for defining cellular phenotypes were based on consensus guidelines and large-scale cohort studies. In addition, the proposed “cellular phenotype” classification (neutrophilic, eosinophilic, lymphocytic, macrophage, and mixed granulocytic phenotypes) is supported by cohort studies across diverse populations (39) (11, 12). Classification details for different phenotypes are listed below:

- Neutrophilic Phenotype: Sputum neutrophils > 60% of total inflammatory cells, driven by neutrophil activation and NET formation (39).
- Eosinophilic Phenotype: Sputum eosinophils ≥ 3% or blood eosinophils ≥ 300/μL, together with type 2 cytokine upregulation (39–41).
- Lymphocytic Phenotype: No unified definition exists. This phenotype is characterized by CD8+ T-cell infiltration in airways, lung parenchyma, bronchoalveolar lavage (BAL) fluid, and sputum. Th1/Th17 imbalance (elevated IFN-γ/interleukin-17) and reduced regulatory T-cell (Treg) function are core features (42, 43).
- Macrophage Phenotype: No unified definition exists. Key features include macrophage infiltration in airways, lung parenchyma, BAL fluid, and sputum, with abnormal CD206 and CD86 expression, which correlates with impaired phagocytosis and M1/M2 imbalance (12, 44, 45).
- Mixed Granulocytic Phenotype: Concurrent elevation of ≥2 cell types (e.g., neutrophils + eosinophils), contributing to severe airflow limitation (39, 46).

This classification system is supported by evidence that each phenotype correlates with distinct inflammatory pathways, treatment implications, and clinical outcomes (47–49).

### 4.1 Neutrophilic phenotype

The neutrophilic phenotype is the most common airway inflammatory cellular subtype in COPD patients (39, 50). A Chinese study reported a prevalence of 58% of this phenotype in 895 COPD patients (39). Its defining feature is increased neutrophil infiltration, which correlates with the airway inflammation severity (51, 52).

In patients with neutrophilic phenotype COPD, activated neutrophils can cause significant tissue damage and airway remodeling (11, 53). The key pathological characteristics of this phenotype include activated neutrophils (54), enhanced migratory capacity (55), delayed apoptosis, and excessive NET formation (11, 52). Activated neutrophils release large quantities of toxic molecules, such as neutrophil elastase and reactive oxygen species (ROS) (56, 57). They also highly express chemokine receptors (CXCR1/2), driving their recruitment to the airways (11). Concurrently, elevated levels of anti-apoptotic factors (e.g., granulocyte-macrophage colony-stimulating factor [GM-CSF], granulocyte colony-stimulating factor [G-CSF]) inhibit neutrophil apoptosis, perpetuating inflammation (58). Dysregulation of apoptosis, including delayed neutrophil apoptosis and impaired clearance of apoptotic cells by macrophages, is a recognized contributor to chronic inflammation and tissue injury in COPD (59, 60). High-mobility group box 1 (HMGB1), via Toll-like receptor 4 (TLR4) signaling, enhances neutrophil recruitment and neutrophil extracellular trap (NET) formation (61). The latter releases DNA mesh structures and cytotoxic proteins, exacerbating tissue injury, chronic inflammation, and bacterial colonization (11).

#### 4.1.1 Therapeutic strategies and research directions for the neutrophilic phenotype

The key therapeutic strategies for neutrophilic phenotype COPD include:

1. Neutrophil recruitment and activation blockage. This can be achieved through the use of chemotaxis inhibition CXCR1/2 antagonists (11, 62) and activation and migration inhibition (LTB4 inhibitors) (63), thereby reducing overall neutrophil recruitment and activation.
2. Inhibiting the release of toxic mediators. Neutrophil elastase inhibitors, such as brensocatib, have shown promise in this regard (64). A phase III trial demonstrated a significant reduction in clinical exacerbation frequency and improved lung function among patients with bronchiectasis, and ongoing studies are evaluating its efficacy with the neutrophilic type of COPD (64). In addition, antioxidants such as N-acetylcysteine can scavenge ROS to reduce NET formation, which might ameliorate the progression of this COPD subtype (64).
3. Promotion of NET clearance or apoptosis reduction. This approach involves the application of NET clearance agents (11) or Bcl-2 inhibitors to facilitate the programmed cell death of neutrophils (65).
4. Modulation of metabolic and cytokine signaling pathways. Cytokines targeting these pathways are currently under investigation, including anti-interleukin-17/interleukin-23 biologics, anti-interleukin-33 antibodies, and anti-TSLP antibodies (50, 66).

### 4.2 Eosinophilic phenotype

Approximately 25%–40% of patients with eosinophilic COPD exhibit an eosinophilic inflammatory phenotype, resembling the patterns observed in asthma (41, 67). This phenotype is characterized by elevated eosinophil counts in blood (≥300/μL) or sputum (≥3%), alongside upregulated cytokines, such as interleukin-5 (40), interleukin-4, and interleukin-13, which drive eosinophil differentiation, recruitment, and survival (11, 68).

The eosinophilic COPD phenotype involves type 2 inflammation driven by both Th2 cells (antigen-dependent, via T-cell receptor activation) and group 2 innate lymphoid cells (ILC2s) and epithelial alarmins (TSLP, interleukin-33), which drive interleukin-5/interleukin-13 secretion independently of atopy. This explains why 28%–32% of patients with eosinophilic COPD lack allergic features (11, 69). Transcriptomic profiling has confirmed that airway eosinophilia (>1% in BAL) is correlated with upregulated type 2 gene signatures (e.g., interleukin-13, mast cell activation), which are suppressed by the administration of inhaled corticosteroids (ICS) (70). Activated eosinophils exacerbate airway inflammation, mucus hypersecretion, and remodeling through the secretion of toxic granule proteins (e.g., eosinophil cationic protein [ECP], major basic protein [MBP]), and pro-inflammatory mediators, such as leukotriene C4 (69).

#### 4.2.1 Therapeutic strategies and research directions for the eosinophilic phenotype

The key therapeutic strategies for eosinophilic phenotype COPD include:

1) Inhaled corticosteroids (ICS). ICS suppress type 2 inflammation and reduce exacerbations, with greater efficacy in patients with blood eosinophil counts ≥ 300/μL (12, 70). However, long-term use could cause an increased risk of pneumonia, particularly in smokers (71).

2) Biologic agents:

- Anti-interleukin-5 pathway therapies. Mepolizumab (anti-interleukin-5) was reported to reduce annualized moderate-to-severe exacerbations by 21% (rate ratio = 0.79, 95% confidence interval 0.66–0.94, *P* = 0.01) in patients with blood eosinophil counts ≥ 300/μL (MATINEE trial, NCT04133909), with a consistent benefit observed in non-allergic subgroups—supporting interleukin-5-driven eosinophilia independent of adaptive allergies (72). However, this single-cytokine targeted approach demonstrated modest efficacy compared to broader type 2 pathway blockade (11, 73). Another study reported that benralizumab (anti-interleukin-5 receptor α antibodies) depleted eosinophils and lowered the exacerbation risk in “pure type 2” subgroups (74). These agents are preferred for patients with isolated eosinophilic inflammation (low neutrophilic markers).
- Broad type 2 inhibition. It was found that dupilumab (anti-interleukin-4Rα, blocking interleukin-4/interleukin-13) could significantly reduce exacerbations by 34% (rate ratio 0.66, 95% confidence interval 0.54–0.82, P<0.001. NOTUS trial) and improve prebronchodilator FEV_1_ by 82 mL at week 12 in patients with blood eosinophil counts of ≥300/μ (68). This aligned with the BOREAS trial, where dupilumab reduced exacerbations by 30%, particularly in patients with higher fractional exhaled nitric oxide (FeNO ≥20 ppb), a marker of active type 2 inflammation (11). This broad type 2 inhibition was more effective than single-cytokine blockers in patients with elevated FeNO (≥20 ppb) or comorbid type 2 conditions (e.g., chronic rhinosinusitis with nasal polyps) (75).

3) Combination therapy. ICS combined with long-acting bronchodilators could balance the anti-inflammatory effects and symptom relief (76). The use of ICS + long-acting beta-agonist (LABA)/long-acting muscarinic antagonist (LAMA) remains foundational, but add-on biologics (e.g., dupilumab) are recommended for patients with persistent exacerbations despite triple therapy (71).

4) Biomarker-guided-subtyping-refined therapy. Higher baseline blood eosinophils and FeNO can predict a better response to dupilumab (75). However, blood eosinophils are weakly correlated with airway type 2 activity, highlighting the need for combined biomarkers (e.g., FeNO, pulmonary and activation-regulated chemokine [PARC]) to identify “type 2-high” patients (70, 71). Upstream alarmins (TSLP, interleukin-33) are also emerging therapeutic targets in trials, addressing mixed subtypes where single-cytokine blockade is less effective (73). Therefore, a dynamic assessment of combined biomarkers—including blood eosinophils, FeNO, and serum eotaxin-3—can provide more accurate treatment targeting than eosinophils alone. For example, the response to dupilumab is stronger in patients with a baseline FeNO ≥ 20 ppb (70, 75). Treatment adjustments may be made based on the dynamic eosinophil levels (51, 77).

Future directions include the development of novel targeted agents (e.g., TSLP inhibitors) to precisely regulate eosinophilic-driven inflammation while minimizing therapeutic adverse effects (73, 78). In addition, cellular therapies are under investigation, with stem cell-based approaches to modulate immune responses, and potentially offer a new treatment regimen for eosinophil-phenotype COPD (79).

### 4.3 Lymphocytic phenotype

Patients with the lymphocytic phenotype COPD are characterized by a Th1/Th17 immune imbalance (42). This manifests as an abnormal expansion of CD8+ T cells and γδ T cells in lung tissue, with subsequent IFN-γ, interleukin-17, and interleukin-22 secretions that drive neutrophilic inflammation and airway remodeling. Concurrently, impaired regulatory T-cell (Treg) function—marked by a reduced FoxP3 expression—minimizes the anti-inflammatory capacity (80). Additional pathogenesis, such as B-cell activation (e.g., autoantibody production) (22) and tertiary lymphoid structure formation (81), can promote chronic inflammation and the progression of emphysema. Cytotoxic T cells contribute directly to alveolar epithelial damage via the release of perforin and granzyme, creating an “autoimmune-like” pathological profile (82). In induced sputum of COPD patients, CD8+ T cells not only increase in number but also express high levels of perforin and exhibit enhanced cytotoxic activity (83, 84).

#### 4.3.1 Therapeutic strategies and research directions for the lymphocytic phenotype

The key therapeutic strategies for the lymphocytic phenotype COPD include:

1. Targeting cytokine pathways. Anti-interleukin-17/interleukin-22 monoclonal antibodies can suppress Th17 inflammation. This treatment approach has been validated in immune-mediated diseases, such as psoriasis, psoriatic arthritis, ankylosing spondylitis, and asthma (85).
2. Enhancing Treg function. Low-dose interleukin-2 or vitamin D supplementation has been shown to promote Treg activity and thereby restore immune tolerance, potentially offering benefits with the lymphocytic COPD phenotype (86, 87).
3. Inhibiting CD8+ T-cell cytotoxicity. Janus kinase (JAK) inhibitors block IFN-γ signaling, a pathway confirmed in Th1/Tc1-dominated diseases, such as rheumatoid arthritis and psoriasis (88).
4. Modulating B-cell activation. Bruton’s tyrosine kinase (BTK) inhibitors could reduce the production of autoantibodies, as demonstrated in rheumatoid arthritis and systemic lupus erythematosus (89).
5. Repurposing existing drugs. Phosphodiesterase-4 (PDE4) inhibitors (e.g., roflumilast) indirectly suppress T-cell proliferation (90). Novel immunomodulators (e.g., anti-OX40L antibodies) may block T-cell co-stimulation. Also, immune checkpoint modulators [e.g., programmed death-1 (PD-1)/programmed death-ligand 1 (PD-L1) inhibitors] are under investigation with an aim to reverse T-cell exhaustion.
6. Cellular therapy. Stem cell-based approaches targeting lymphocyte subsets can repair immune dysfunction and alleviate COPD symptoms (79).

Future directions include single-cell sequencing, which could help define patient-specific lymphocyte subpopulations, enabling precision combination therapies that balance anti-inflammatory efficacy with the risk of infection.

### 4.4 Macrophage phenotype

Macrophages are involved in the immune responses and pathogenesis in COPD patients (91). Under normal conditions, macrophages clear pathogens and apoptotic cells via phagocytosis. However, in patients with COPD, macrophage function is often impaired, reducing the clearance efficiency and exacerbating inflammatory responses (47, 92). The number of macrophages is significantly increased in the small airways of patients with COPD, correlated with the disease severity, where higher macrophage counts correspond to a more advanced pathology (12, 93). These cells release cytokines and chemokines (e.g., CCL-2, CCL-3) that amplify inflammation and recruit additional inflammatory cells to the lungs (94). Furthermore, macrophages play a critical role in tissue remodeling by secreting cytokines and growth factors, such as matrix metalloproteinases and ROS, altering neighboring cell activity and promoting lung tissue repair and regeneration (44, 47). Thus, the ‘macrophage phenotype’ refers to the dominant functional states rather than discrete subsets, acknowledging the spectrum of activation states observed *in vivo*.

Traditionally, macrophages are commonly classified into M1 (pro-inflammatory) and M2 (anti-inflammatory/reparative) subtypes, which may oversimplify their complexity in COPD. As highlighted by Murray et al. (95), macrophages in COPD often exhibit intermediate or plastic phenotypes, with the co-expression of both M1 and M2 markers (e.g., simultaneous expression of TNF-α and interleukin-10) under dynamic environmental stimuli (e.g., cigarette smoke, microbial products). This plasticity limits strict M1/M2 categorization and underscores the need for nuanced subtyping via single-cell profiling. Meanwhile, macrophages in COPD often exhibit an intermediate phenotype, combining the pro-inflammatory features of M1 and anti-inflammatory characteristics of M2 (44, 96).

#### 4.4.1 Therapeutic strategies and research directions for the macrophage phenotype

The key therapeutic strategies for macrophage phenotype COPD include:

1. Glucocorticoids (e.g., prednisone). Glucocorticoids can suppress pro-inflammatory cytokine production, reducing M1 macrophage activity (97).
2. Small-molecule drugs (e.g., phosphatidylinositol 3-kinase [PI3K]/mammalian target of rapamycin [mTOR] inhibitors). These drugs have demonstrated potential in modulating macrophage metabolism and the phenotype. By altering metabolic pathways, these drugs promote M1-to-M2 phenotypic shifts, attenuating COPD-related inflammation (98).
3. Phagocytic function enhancement. Pro-resolving mediators (e.g., resolvin D1) can improve the clearance of apoptotic cells (48).
4. M2 polarization promotion. Interleukin-10 analogs or peroxisome proliferator-activated receptor gamma (PPARγ) agonists (e.g., rosiglitazone) can restore anti-inflammatory and reparative functions (48, 49).

### 4.5 Mixed granulocytic phenotype

The mixed granulocytic COPD phenotype is characterized by synergistic activation and complex interactions among multiple inflammatory cells, including neutrophils, macrophages, lymphocytes, and eosinophils. A Chinese study of 895 COPD patients reported the prevalence of this phenotype was 32.6% (39). These patients often present with more severe airflow limitation and dyspnea (39), likely due to irreversible airway damage from intensified airway inflammation and aberrant cytokine release (46). During acute exacerbations, patients with mixed granulocytic COPD often demonstrate elevated eosinophil and neutrophil counts. This phenotype also illustrates that cellular patterns are not mutually exclusive but can overlap, forming a continuum of inflammatory states.

#### 4.5.1 Therapeutic strategies and research directions for the mixed granulocytic phenotype

Given the involvement of multiple cell types, the core therapeutic strategy for this phenotype is the use of combinatorial approaches targeting multi-cellular inflammatory networks, such as:

1. Combination pharmacotherapy. ICS combined with long-acting bronchodilators (LABA/LAMA) can broadly suppress inflammation and improve airflow limitation (12).
2. Multi-pathway modulations. Dual interleukin-5/interleukin-17 inhibitors (72, 73) and JAK inhibitors (99) target neutrophils, eosinophils, and lymphocytes to synergistically inhibit the inflammatory network (100).
3. Stem cell therapy. Exogenous stem cells differentiated into lung-specific phenotypes can promote tissue repair and functional recovery (79).
4. Molecular-targeted therapy. Novel agents developed through molecular profiling (e.g., signaling pathways, transcription factors) can enable a precision modulation of cellular functions for individualized treatment (101).

**Figure 1:**
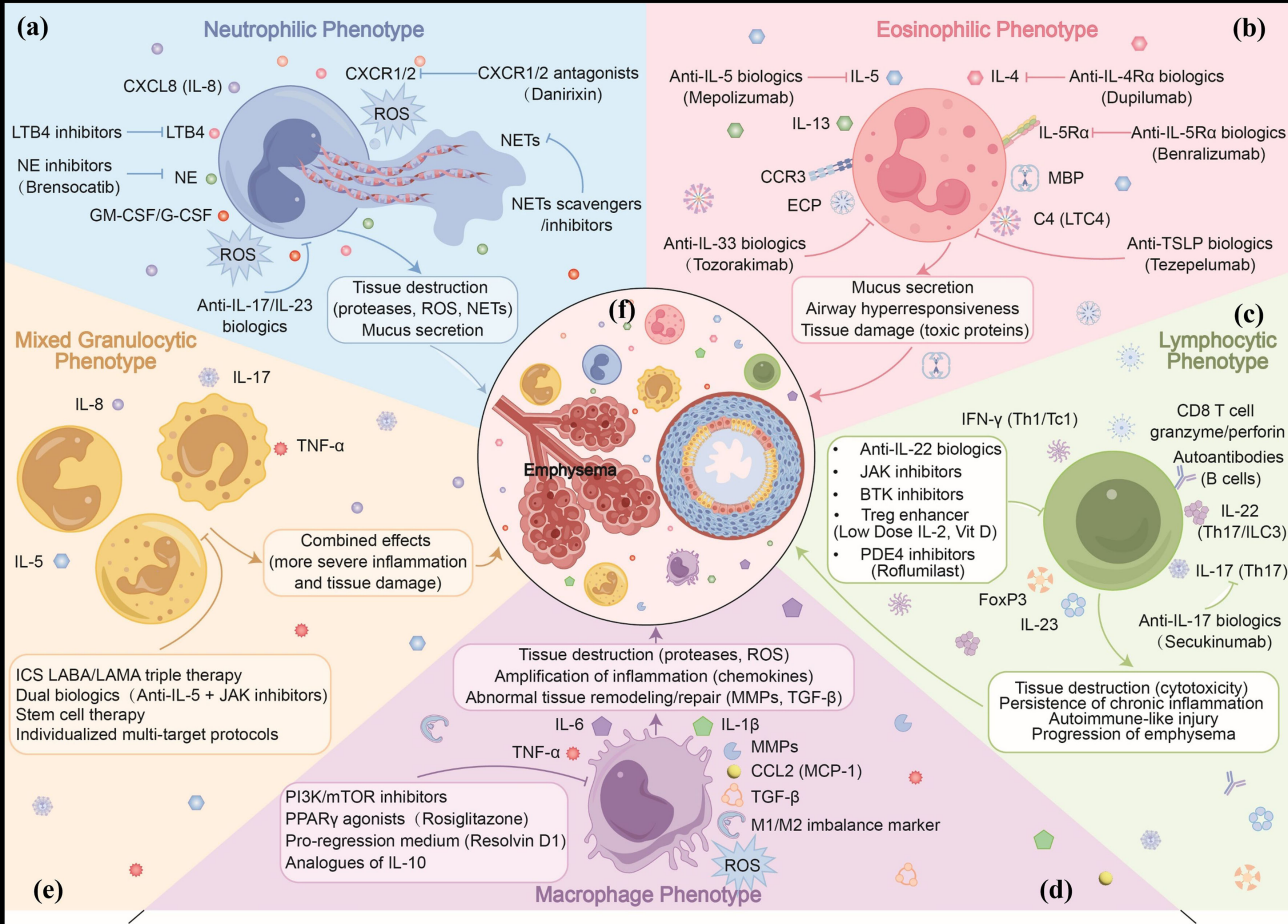
Cellular endotyping in COPD: From molecular pathogenesis to phenotype-targeted therapeutics. **(a)** Neutrophilic phenotype: Marked by CXCL8, NE, ROS, and NET formation, causing protease-driven tissue injury. Candidate interventions include CXCR1/2 antagonists, NE inhibitors, and LTB4 blockade. **(b)** Eosinophilic phenotype: Driven by IL-4, IL-5, and IL-13, with eosinophil degranulation promoting airway remodeling. Candidate biologics target IL-5/IL-5Rα, IL-4Rα, IL-33, and TSLP. **(c)** Lymphocytic phenotype: Characterized by IFN-γ-driven CD8+ cytotoxicity, Th17/ILC3 cytokines, and impaired Treg function. Potential targets include anti-IL-17, JAK/BTK inhibitors, and PDE4 inhibition. **(d)** Macrophage phenotype: Defined by inflammatory cytokine release, M1/M2 imbalance, and dysregulated repair. Proposed targets: PI3K/mTOR pathway and PPARγ. **(e)** Mixed granulocytic phenotype: Features overlapping inflammation involving ≥2 cell types. Managed with multi-target strategies including triple therapy and combination biologics. **(f)** Central panel: Shared downstream outcomes of airway injury, parenchymal destruction, and emphysema. CXCL8, C-X-C motif chemokine ligand 8; NE, neutrophil elastase; ROS, reactive oxygen species; NETs, neutrophil extracellular traps; LTB4, leukotriene B4; IL, interleukin; IL-5Rα, IL-5 receptor alpha; IL-4Rα, IL-4 receptor alpha; TSLP, thymic stromal lymphopoietin; IFN-γ, interferon-gamma; Th17, T helper 17; ILC3, group 3 innate lymphoid cell; Treg, regulatory T cell; JAK, Janus kinase; BTK, Bruton’s tyrosine kinase; PDE4, phosphodiesterase 4; PI3K, phosphoinositide 3-kinase; mTOR, mechanistic target of rapamycin; PPARγ, peroxisome proliferator-activated receptor gamma.

**Figure 1** illustrates the pathogenesis of COPD and the corresponding therapeutic strategies based on cellular phenotypes. These therapeutic strategies (**Table 1**) are currently under rigorous clinical evaluation. However, early-phase trials have demonstrated promising efficacy in modulating cellular drivers, while phase III studies will establish their impacts on exacerbation reduction.

**Table 1:** Registered clinical trials targeting cellular phenotypes in COPD patients.

Cellular phenotype	Cellular target	Drug/intervention	Mechanism	Phase	NCT identifier	Status
Neutrophilic (>60% sputum neutrophils)	Neutrophils	Danirixin	Selective CXCR2 antagonist	II	NCT03250689	Terminated
Eosinophilic (≥3% sputum or ≥300/μL blood eosinophils)	Eosinophils	Tozorakimab	Monoclonal antibody (anti-interleukin-33)	III	NCT06897748	Recruiting
	Eosinophils	Benralizumab	Monoclonal antibody (anti-IL5RA)	III	NCT04053634	Active, not recruiting
	Eosinophils	Mepolizumab	Monoclonal antibody (anti-IL5)	III	NCT02105961	Completed (2017)
	Eosinophils	Mepolizumab	Monoclonal antibody (anti-IL5)	III	NCT04133909	Completed (2024)
	Eosinophils	Mepolizumab	Monoclonal antibody (anti-IL5)	III	NCT02105948	Completed (2017)
	Eosinophils	Dupilumab	Anti-IL-4Rα mAb	III	NCT03930732	Completed (BOREAS)
Lymphocytic	lymphocyte	CNTO 6785	Anti-IL-17A mAb	II	NCT01966549	Completed (primary endpoint not met)
	lymphocyte	KN-002	Inhaled pan-JAK inhibitor	Ib	NCT05006521	Completed

*For the lymphocytic and macrophage phenotypes, no agents have yet entered phase III trials in COPD. CNTO 6785 did not meet its primary efficacy endpoint in a phase II study, and JAK inhibition remains at an early investigative stage. Macrophage-targeted therapies have not yet reached registered interventional trials. These phenotypes therefore remain areas of ongoing research without established clinical-stage interventions.

## 5 Stepwise diagnostic pathway for COPD cellular phenotype identification

**Figure 2:**
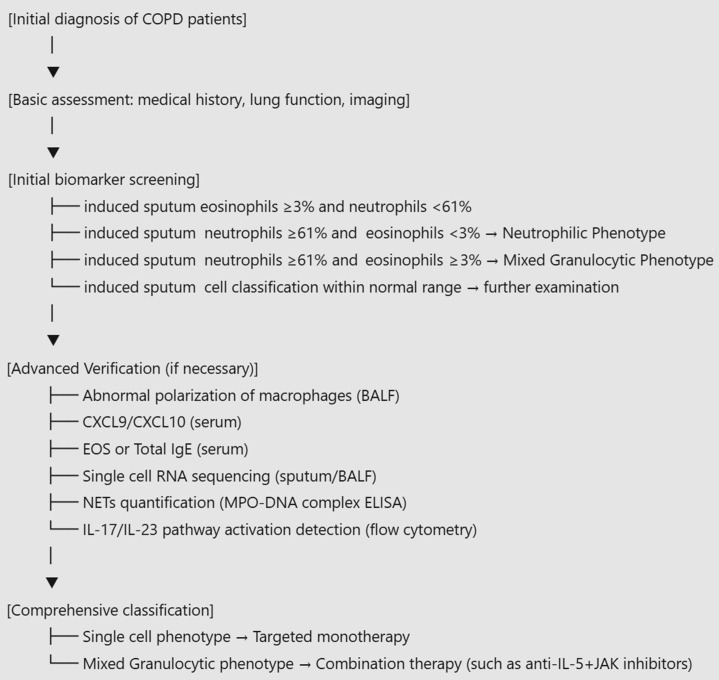
COPD cellular phenotype diagnostic pathway. This figure outlines a stepwise pathway for stratified diagnosis and treatment of COPD based on cellular phenotypes. After initial evaluation—including medical history, lung function testing and imaging—patients undergo first-line biomarker screening using induced sputum cell differentials to identify eosinophilic, neutrophilic or mixed granulocytic inflammatory patterns (e.g., eosinophils ≥3% and/or neutrophils ≥61%). A neutrophilic phenotype is assigned when sputum neutrophils are ≥61% with eosinophils <3%, whereas a mixed granulocytic phenotype is assigned when sputum neutrophils are ≥61% with eosinophils ≥3%. If sputum cell profiles fall within the normal range or additional confirmation is required, advanced verification can be performed using complementary biomarkers and multi-omics approaches, such as assessment of macrophage polarization in bronchoalveolar lavage fluid (BALF), serum CXCL9/CXCL10, blood eosinophil counts or total IgE, single-cell RNA sequencing in sputum/BALF, NET quantification by MPO–DNA complex ELISA, and evaluation of IL-17/IL-23 pathway activation by flow cytometry. Comprehensive classification then informs phenotype-matched therapy, with targeted monotherapy for single-cell phenotypes and combination strategies more often considered for mixed phenotypes. COPD, chronic obstructive pulmonary disease; BALF, bronchoalveolar lavage fluid; EOS, eosinophils; IgE, immunoglobulin E; NETs, neutrophil extracellular traps; MPO, myeloperoxidase; ELISA, enzyme-linked immunosorbent assay; IL, interleukin; JAK, Janus kinase.

We propose a four-tiered approach as follows (**Figure 2**): 1) Conduct initial biomarker screening to identify the dominant cellular patterns based on sputum or blood cell type assessments; 2) Perform multi-omics validation for indeterminate cases; 3) Assign a consensus phenotype based on integrated data; and 4) Adopt dynamic therapy using longitudinal biomarkers. This approach shifts COPD diagnosis and treatment from symptom clusters to mechanism-based classification, from static to dynamic monitoring, and from empirical to precision targeting.

## 6 Limitations in the application of cellular phenotypes

Although the cellular phenotype COPD classification offers a more pathogenetic framework, several significant challenges might hinder its immediate and widespread clinical translation:

- Standardization and definition gaps. Clinical phenotype classification relies heavily on imaging or symptomatic features, with little consideration of the molecular mechanisms. Some clinical phenotypes exhibit overlapping definitions or involve subjective criteria. Similarly, certain cellular phenotypes—particularly lymphocytic and macrophage subtypes—lack universally accepted definitions and standardized biomarker thresholds (e.g., for blood eosinophils, NETs), creating inconsistency across studies, thus complicating clinical adoption. Clinical phenotyping in particular has focused largely on superficial differences in clinical manifestations without rigorous correlation with underlying pathophysiology, limiting its value for guiding mechanism-based treatment.
- Dynamic heterogeneity and longitudinal data: Cellular phenotype studies to date have predominantly used cross-sectional data, lacking robust evidence on dynamic evolution over time or long-term clinical outcomes. The stability of a patient’s cellular phenotype and its transitions during stability, exacerbation, or in response to therapy remains poorly understood. Notably, these phenotypes do not represent disease stages. Extracellular trap (ET) formation, for example, may be driven predominantly by macrophages or neutrophils during stable COPD, but shifts to a predominantly neutrophil-driven process during exacerbations—reflecting a dynamic transition rather than a fixed phase.
- Complexity of mixed phenotypes. Management of the mixed granulocytic phenotype remains dependent on empirical drug combinations, with limited high-efficacy strategies for integrated multi-pathway intervention. The intricate cellular crosstalk in these patients presents a major challenge and complicates drug selection.
- Validation and accessibility of tools. Novel targeted agents (e.g., CXCR1/2 antagonists, interleukin-17 inhibitors) require large-scale, well-designed trials to confirm their efficacy and safety in specific COPD subpopulations, alongside rigorous cost-benefit analyses. Furthermore, multi-omics profiling and single-cell analyses—crucial for refining these phenotypes—are not yet routinely accessible in most clinical settings.

## 7 Future research directions

To overcome current limitations and promote precision medicine in COPD patients, future research should focus on:

- Deep phenotyping via multi-omics: Leveraging single-cell multi-omics approaches (e.g., spatial transcriptomics, proteomics) will enable a comprehensive mapping of immune cell signatures and their spatial relationships within the lung tissue. This strategy will help clarify the functional heterogeneity within broadly defined cellular phenotypes and identify novel, therapeutically targetable subpopulations.
- Longitudinal cohort studies. Longitudinal studies that follow patients over time using dynamic biomarkers, such as epigenomics and metabolomics, will clarify the natural history and evolution of cellular phenotypes. These studies can also explore the predictive value of such biomarkers for COPD progression and the treatment response.
- Advanced predictive modeling. Machine learning and artificial intelligence could be applied to integrate multidimensional data from imaging radiomics, plasma proteomics, and clinical variables to develop robust models that can predict the phenotypic assignment and treatment outcomes.
- Adaptive and umbrella clinical trials. Adaptive clinical trials (e.g., MASTERPLAN-COPD) should be designed to test omics-guided therapy strategies. These trials can enable the real-time assignment of patients to treatments based on their evolving cellular profiles, thereby advancing precision medicine in COPD research.

## 8 Conclusion

In a heterogeneous COPD patient population, effective management requires moving beyond any single classification approach. The evolution from clinical to cellular phenotyping has deepened our understanding of COPD heterogeneity, and the next step lies in integrating clinical presentation, cellular drivers, and multi-omics signatures into a multidimensional framework. Such integration is key to improving patient outcomes and advancing toward genuine precision medicine. Cellular phenotypes, based on disease driver cells and pathways, can guide targeted therapies (biologics, small molecules) and their rational combinations (ICS + immunomodulators). Clarifying COPD heterogeneity via cellular typing, developing multi-pathway therapies for mixed phenotypes, and implementing dynamic biomarker-guided algorithms may fundamentally shift management from symptom control to pathogenesis modification. Bridging the gap between molecular mechanisms and clinical application remains both the central challenge and the major opportunity for the coming decade of COPD research.
